# Bacterial, but not fungal, communities show spatial heterogeneity in European beech (*Fagus sylvatica L*.) deadwood

**DOI:** 10.1093/femsec/fiad023

**Published:** 2023-03-11

**Authors:** Jason Bosch, Ema Némethová, Vojtěch Tláskal, Vendula Brabcová, Petr Baldrian

**Affiliations:** Laboratory of Environmental Microbiology, Institute of Microbiology of the Czech Academy of Sciences, Vídeňská 1083, 142 20 Prague, Czechia; Laboratory of Environmental Microbiology, Institute of Microbiology of the Czech Academy of Sciences, Vídeňská 1083, 142 20 Prague, Czechia; Laboratory of Environmental Microbiology, Institute of Microbiology of the Czech Academy of Sciences, Vídeňská 1083, 142 20 Prague, Czechia; Laboratory of Environmental Microbiology, Institute of Microbiology of the Czech Academy of Sciences, Vídeňská 1083, 142 20 Prague, Czechia; Laboratory of Environmental Microbiology, Institute of Microbiology of the Czech Academy of Sciences, Vídeňská 1083, 142 20 Prague, Czechia

**Keywords:** bacteria, deadwood, ecology, fungi, microbial communities, temperate forest, wood decay

## Abstract

Deadwood decomposition and other environmental processes mediated by microbial communities are generally studied with composite sampling strategies, where deadwood is collected from multiple locations in a large volume, that produce an average microbial community. In this study, we used amplicon sequencing to compare fungal and bacterial communities sampled with either traditional, composite samples, or small, 1 cm^3^ cylinders from a discrete location within decomposing European beech (*Fagus sylvatica L*.) tree trunks. We found that bacterial richness and evenness is lower in small samples when compared to composite samples. There was no significant difference in fungal alpha diversity between different sampling scales, suggesting that visually defined fungal domains are not restricted to a single species. Additionally, we found that composite sampling may obscure variation in community composition and this affects the understanding of microbial associations that are detected. For future experiments in environmental microbiology, we recommend that scale is explicitly considered as a factor and properly selected to correspond with the questions asked. Studies of microbial functions or associations may require samples to be collected at a finer scale than is currently practised.

## Teaser

Bacterial communities show different patterns of diversity depending on sampling scale, whereas fungal communities show even distribution throughout deadwood.

## Introduction

Forests cover 35% of the land area of Europe with deadwood accounting for, on average, 1.5 m^3^/ha or 7% of the total carbon pool (FOREST EUROPE 2020). Deadwood decomposition is initiated by fungi that are able to colonize deadwood due to their filamentous growth, lower nitrogen requirements, and enzymes necessary to digest recalcitrant molecules such as lignin and cellulose. This initial colonization appears to be primarily stochastic and depends on the species either already present in the living tree or recruited from the soil (Baldrian et al. 2016, Baldrian 2017, Hagge et al. 2019). The fungi grow through the deadwood, encounter hyphae from other species and compete, eventually establishing zones of control from which other fungal species are excluded (Boddy 2000, Heilmann-Clausen and Boddy 2005, Hiscox and Boddy 2017, O'Leary et al. 2018). During decomposition, simpler sugars are freed, which allow prokaryotes to grow in the deadwood and provide nitrogen and other compounds (Hoppe et al. 2014, Tláskal et al. 2021a).

Ecological interactions, such as those involved in deadwood degradation, can not be fully understood without taking scale into account (Ladau and Eloe-Fadrosh 2019). At a large scale, measured in kilometres, we might recognize forests as a single type of environment but, inside a forest, there are multiple smaller environments, including deadwood, soil, water, rock surfaces, and air (Baldrian 2016). Even in these smaller environment, such as soil, there can be a large amount of heterogeneity at the metre (Štursová et al. 2016) or centimetre (O’Brien et al. 2016) scales, leading to very different microbial communities and biological processes existing just a short distance from one another. Furthermore, even samples taken several centimetres apart are more likely to represent meta-communities than the actual microbial communities wherein real interactions take place (Cordero and Datta 2016). This is supported by recent work showing that regularly mixing soil samples reduces the microbial richness, emphasizing the importance of divergent microbial communities that exist on the micro scale (West and Whitman 2022).

Organisms interact and influence one another; invertebrates influence fungal communities by influencing the outcome of fungal–fungal interactions (Crowther et al. 2011) and spreading fungi to new environments (Eskalen et al. 2013, Paap et al. 2018, Seibold et al. 2019). In turn, fungi influence bacteria in ways that includes antagonistic interactions (Deveau et al. 2018, Carmona-Hernandez et al. 2019), providing niches for bacterial growth (Boer et al. 2005, Xiong et al. 2022), facilitating bacterial movement through the environment (Christofides et al. 2020, Xiong et al. 2022) and as a carbon source (Brabcová et al. 2016). While the exact nature of the interaction between fungi and bacteria will change depending on the current environmental state (Zhang et al. 2016), we expect that, at small scales, volumes of deadwood will be controlled by a limited number of dominant fungi and those fungi will be associated with a specific bacterial community. Small-scale sampling may thus allow us to analyse the nature of pairwise interactions between the dominant fungus and individual bacterial taxa.

In this study, we compared the microbial communities of deadwood objects from the Žofín forest—an unmanaged, mixed, European forest—sampled in the form of approximately 1 cm^3^ cylinders to existing composite samples of pooled sawdust from multiple drillings that were collected to represent the microbial community within a whole deadwood object. As bacterial community composition is driven by environmental factors, we hypothesized that fine-scale sampling may reveal the deadwood microenvironment whereas composite sampling will contain communities from several microenvironments. Given current knowledge of fungal zones in deadwood, we hypothesized that fine-scale samples would show lower levels of fungal diversity due to the restricted sampling size and would be dominated by one or two main fungal taxa.

## Methodology

### Sampling, DNA extraction, and sequencing

The study was conducted in the Žofínský Prales National Nature Reserve (48° 39′ 57'' N, 14° 42′ 24'' E; Figure S1, Supporting Information) in the south of the Czech Republic, which is unmanaged and protected since 1838. Composite samples were harvested from European beech (*Fagus sylvatica L*.) deadwood objects (decomposing tree trunks) in November 2016 and consisted of pooled sawdust from drilling four holes per log using a 10 mm diameter drill bit. A detailed description of the selected logs, sampling process and sequencing can be found in our previous publications (Tláskal et al. 2021a,b).

In June 2019, 10 logs, five of which were estimated to have died and fallen between 1997 and 2008 and five of which were estimated to have died and fallen between 2008 and 2013, were resampled to acquire fine-scale samples. For each log, three drillings were made with a hollow, 10 mm diameter drill bit to a depth of ∼10 cm. As compression of the wood occurred during the drilling process, individual judgement was used to remove a 1-cm^3^ cylinder from the deadwood core. Each cylinder was ground into powder with a mortar and pestle before being processed in the same manner as the composite samples.

Total DNA was extracted in triplicate from 200 mg of powdered deadwood using a NucleoSpin soil kit (Macherey-Nagel). The fungal ITS2 region was amplified using barcoded gITS7 (5′-GTGAATCATCGAATCTTTG-3′) and ITS4 (5′-TCCTCCGCTTATTGATATGC-3′) primers (Ihrmark et al. 2012) and the bacterial 16S rRNA gene was amplified using barcoded 515F (5′-GTGCCAGCMGCCGCGGTAA-3′) and 806R (5′-GGACTACHVGGGTWTCTAAT-3′) primers (Caporaso et al. 2012). PCR amplification was performed with 5 μl of 5x Q5 reaction buffer (New England Biolabs), 5 μl of 5x Q5 high GC enhancer (New England Biolabs), and 0.25 μl Q5 high-fidelity DNA polymerase (New England Biolabs), 1.5 μl of bovine serum albumin (10 mg/ml), 0.5 μl of dNTPs nucleotide mix 10 mM (Bioline), 1 μl of each primer, 9.75 μl of H_2_O, and 1 μl of template DNA. The ITS2 reaction was cycled for 5 min at 94°C, 30 cycles of (30 s at 94°C, 30 s at 56°C, and 30 s at 72°C) and 7 min at 72°C while the 16S reaction was cycled for 4 min at 94°C, 25 cycles of (45 s at 94°C, 60 s at 50°C, and 75 s at 72°C) and 10 min at 72°C. The PCR reactions were purified with a MinElute PCR Purification Kit (Qiagen) and mixed in equimolar amounts according to concentration measured on the Qubit 2.0 Fluorometer (Thermo Fisher Scientific). Sequencing libraries were prepared using the TruSeq PCR-Free Kit (Illumina) according to manufacturer’s instructions and were sequenced on an Illumina MiSeq (2 × 250 bases).

### Preprocessing and ASV calling

The sequencing data was imported and processed with QIIME2 (Bolyen et al. 2019). The quality scores were visualized and used to inform the trimming thresholds. For the bacterial 16S reads, the first 20 base pairs were trimmed from both the forward and reverse reads, forward reads were truncated at length 235 bp and forward and reverse reads were joined with DADA2 (Callahan et al. 2016) during the ASV calling process. In order to maximize the diversity of microbes detected, DADA2 was run with pooled processing (Kleine Bardenhorst et al. 2022). For fungal ITS sequences, only the forward, full-length amplicon sequences were used. The first 20 base pairs of the fungal reads were trimmed and the DADA2 settings were adjusted to allow a maximum of eight expected errors and to truncate reads when quality scores dropped below 8 in accordance with recommendations for DADA2 processing of ITS sequences (Rolling et al. 2022). Once the ASVs had been identified, taxonomy was assigned by a naïve Bayes classifier. The classifier was pretrained on the Silva 138.1 database (Quast et al. 2013, Yilmaz et al. 2014), with reference sequences extracted using the 515F/806R primers, for bacterial sequences and on the UNITE 8.3 database (Nilsson et al. 2019, Kõljalg et al. 2020) for fungi. After processing, 958 457 total bacterial reads were kept with individual samples containing between 6342 and 108 146 reads with a mean of 23 961 reads and a median of 14 585 reads. A total of 1419 637 fungal reads were kept with the number per sample ranging from 9783 to 193 144 with a mean of 35 491 and a median of 21 257 reads.

### Data import and cleaning

All the data was analysed in R 4.2.2 (R Core Team 2022). The following CRAN packages were used to perform the analysis with their versions set by the groundhog package to match the date 2022–09-01: groundhog (Simonsohn and Gruson 2021), vegan (Oksanen et al. 2019), stringr (Wickham 2019), ggplot2 (Wickham 2016: 2), ggsignif (Ahlmann-Eltze 2019), ggrepel (Slowikowski 2021), RcolorBrewer (Neuwirth 2014), gridExtra (Auguie 2017), rnaturalearth (South 2017a), rnaturalearthdata (South 2017b), ggspatial (Dunnington 2021), sf (Pebesma 2018), cluster (Maechler et al. 2021), NbClust (Charrad et al. 2014), ggdendro (Vries and Ripley 2020), ggvenn (Yan 2021), and shipunov (Shipunov et al. 2022). Git packages ggpubfigs (Steenwyk and Rokas 2021) and ggdendroplot (Huber 2021) were also installed through groundhog, however, NetCoMi 1.0.2 (Peschel et al. 2020) was used without groundhog due to installation difficulties. As groundhog does not support BioConductor packages, phyloseq 1.40.0 (McMurdie and Holmes 2013) was also used without groundhog version control.

ASVs with fewer reads than 0.1% of the mean number of reads per sample were removed as these are more likely to contain cross-contaminants. This removed 4097 bacterial and 2327 fungal ASVs. Bacterial ASVs were further filtered to remove ASVs, which were classified as eukaryotic, mitochondrial, chloroplasts, or unassigned at even the domain level. After filtering, 3297 prokaryotic (one archaeal and 3296 bacterial ASVs) and 956 fungal ASVs were retained.

### Diversity metrics

Alpha diversity was measured in terms of the observed number of ASVs (richness), Simpson’s index (1-D) and the Shannon index. In order to eliminate the effects of different library sizes, all samples were rarefied to size of the smallest library. Rarefaction has been criticized as statistically invalid (McMurdie and Holmes 2014) and introducing more biases than it removes (Willis 2019), but was used as pipeline changes were unable to fully eliminate the correlation between diversity and sequencing depth (Kleine Bardenhorst et al. 2022).

Beta diversity was calculated using the weighted Jaccard index and, additionally, with the Euclidean distance of Hellinger-transformed abundances. Ordination plots were constructed by nonmetric multidimensional scaling (NMDS) with beta diversity distances calculated on microbial relative abundance using all ASVs (McKnight et al. 2019).

### Clustering

Clustering of samples was performed using hierarchical clustering of the Jaccard distances and the Ward.D2 method. The optimal number of clusters was the most common number returned from a list of 20 clustering indices. Clusters were bootstrapped with 1000 iterations using the bootstrapped hclust function from the Shipunov R package (Shipunov et al. 2022).

### Association networks

To estimate how sampling scales may influence interpretation of microbial communities, we constructed association networks independently for bacteria and fungi. Taxa with fewer than 60 reads were excluded. These networks were generated using the compositionally aware CCREPE algorithm (Faust et al. 2012). We compared the overall structure of the composite and fine-scale networks with the netCompare function of the NetComi R package (Peschel et al. 2020) and identified the overlap of associations with an edge weight greater than 0.5.

## Results

**Figure 1.**
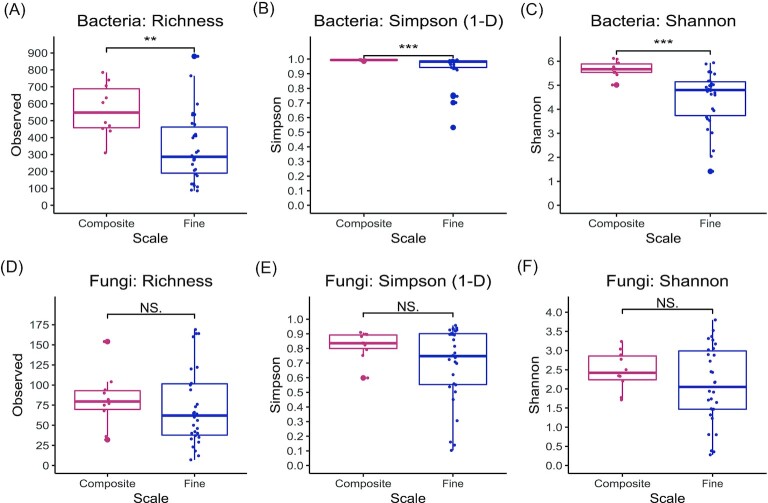
Alpha diversity of deadwood microbial communities. Graphs showing the alpha diversity of bacteria and fungi as measured by either richness (A) and (D), the Simpson index (B) and (E), or the Shannon index (C) and (F). Statistical significance was calculated using the Wilcox test. *P*-values: *** = .001, ** = .01, * = .05, and NS = not significant.

Alpha diversity was assessed through three metrics; ASV richness provides a measure of the number of individual ASVs, which were identified, the Simpson index provides a measure of how evenly distributed the ASVs are and the Shannon index provides a metric, which takes into account both evenness and richness. We observed significantly lower levels of bacterial alpha diversity in the fine-scale samples when compared to the composite samples across all metrics (Fig. 1). In contrast to bacteria, there was no significant difference between composite and fine-scale fungal communities (Fig. 1). Bacterial alpha diversity was considerably higher than fungal diversity, e.g. there were approximately five times as many bacterial ASVs as there were fungal ASVs.

**Figure 2.**
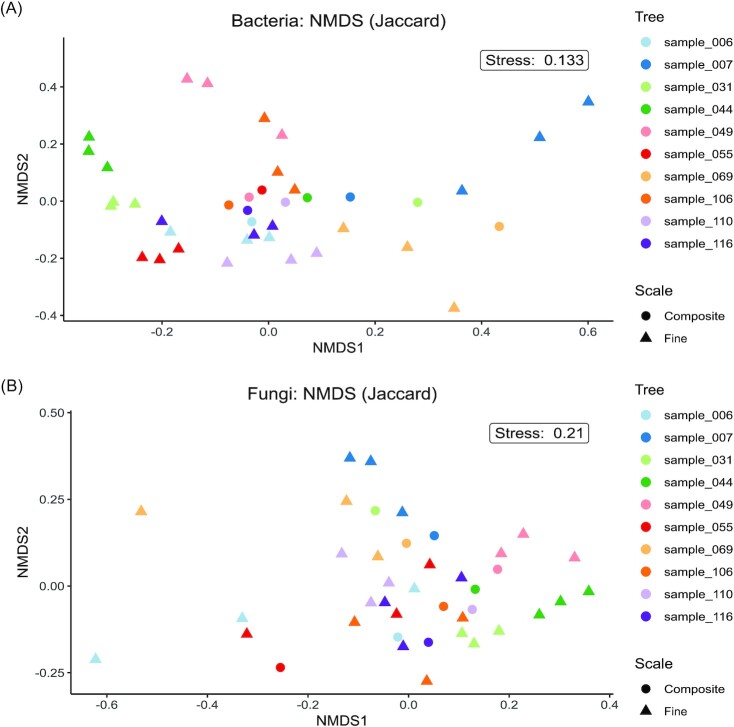
Microbial ordination. NMDS plots of the bacterial and fungal communities based on quantitative Jaccard distances at the ASV level.

Fine-scale samples from each log tended to cluster together on an ordination plot while the majority of the composite samples clustered in the centre of the plot (Fig. 2). This was confirmed by hierarchical clustering analysis, which showed that composite samples generally clustered among one another and not with fine-scale samples from the same deadwood object (Figure S2, Supporting Information). Ordination patterns were robust to the distance metric used (Figure S3, Supporting Information).

**Figure 3.**
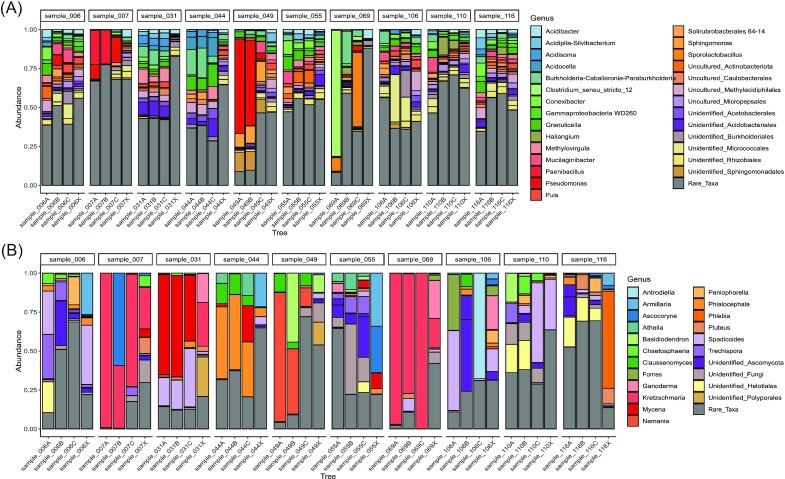
Taxonomic composition. Stacked bar charts showing the taxonomic composition of the fine-scale (bars A, B, and C) and composite (bars X) samples at the genus level for bacteria (A) and fungi (B). Only ASVs with a mean relative abundance greater than 1% are identified, all ASVs with a mean relative abundance less than 1% are included in the grey ‘Rare_Taxa’ category.

The three most common bacterial phyla overall (Figure S4, Supporting Information) were Proteobacteria (42.99%), Actinobacteriota (13.73%), and Acidobacteriota (11.81%). The three most common bacterial genera (Fig. 3) were *Pseudomonas* (3.94%), *Burkholderia–Caballeronia–Paraburkholderia* (3.61%), and *Granulicella* (3.06%). Nearly half of the ASVs (49.24%) belonged to genera, which were detected at a relative abundance of less than 1%. For fungi, ASVs could be assigned to Ascomycota (60.83%), Basidiomycota (34.19%), and unidentified fungi (4.11%) with fewer than 1% belonging to other phyla. The most common fungal genera (Fig. 3) were *Kretzschmaria* (13.97%), *Spadicoides* (8.26%), and *Mycena* (5.42%). As with bacteria, many ASVs (30.23%) belonged to genera which made up less than 1% of the population.

We generated CCREPE association networks from the composite and fine-scale bacterial and fungal data (Figure S5, Supporting Information). Comparison of the networks showed that the composite and fine-scale networks differed for both taxonomic groups with the fine-scale networks showing higher levels of connectivity and lower average dissimilarity with significant differences in clustering. While we observed that a large proportion of associations in the composite networks were also identified in the fine-scale networks (86% for bacteria, 75% for fungi), there were many associations that were specific to either the bulk or fine-scale networks. In the case of the bacterial association networks, the vast majority of fine-scale associations were not detected in the composite network.

## Discussion

### Fine-scale samples show lower diversity than composite samples for bacterial, but not fungal, communities

In line with our hypothesis about bacterial communities, we found fine-scale samples showed significantly lower levels of bacterial diversity compared to composite samples (Fig. 1). Furthermore, the increased community evenness in composite samples suggests that spatial heterogeneity among the bacterial community is obscured when composite samples are formed by pooling sawdust; e.g. combining different bacterial communities results in the increased ASV richness that we observed. We believe that this heterogeneity is caused by differences in the deadwood microenvironment (Moll et al. 2018) but, due the limited sample material, we were unable to test this. This is important for biodiversity surveys as it shows that repeated sampling of the deadwood should result in more ASVs being detected as different microenvironments are sampled.

In contrast to the bacterial results and our expectations, we observed no significant differences in fungal alpha diversity (Fig. 1) between the composite and fine-scale samples. This could be a consequence of the differences in fungal and bacterial biology; fungal hyphae extend throughout the deadwood and nutrients may be transported throughout the hyphal network, allowing filamentous fungi to fully colonize a deadwood object while bacteria are forced to move through the deadwood as nutrients are exhausted or the environmental conditions, e.g. pH, change. This hyphal growth also represents that multcellular fungal structures operate on a much greater physical scale than unicellular prokaryotes and so will be less affected by sampling at different points along the same object. The lack of difference in alpha diversity between the composite and fine-scale fungal further supports the view of complete colonization by a hyphal network and suggests that repeated sampling would not capture any more fungal diversity.

While some samples were dominated by one, or a few, fungal ASVs (e.g. sample_069A-C, Fig. 3), we observed between 5 and 111 (mean: 45.62, median: 43) genus-level fungal ASVs in each sample. Our expectation of a single fungal species dominating a small area of deadwood comes from studies of fungal–fungal interactions on deadwood and the observation of dark interaction zone lines in deadwood (Boddy 2000, Heilmann-Clausen and Boddy 2005, Hiscox and Boddy 2017, O’Leary et al. 2018). These experiments are obviously biased towards those fungi, which grow rapidly and can be cultured. Our amplicon-sequencing results suggest that while certain fungi may compete and exclude one another, the situation in deadwood is more complex and those fungal domains often do not belong to a single fungal species. Alternatively, fungal domains may exclusively belong to a single species but some of those domains may be too small to observe with the naked eye. Technical challenges connected with fine-scale sampling have precluded reaching the necessary spatial resolution to distinguish between these possibilities.

### Microbial heterogeneity must be accounted for in order to identify associations

In most environmental microbiology studies, samples are pooled in order to reduce variation (Alteio et al. 2021) and ensure that the sample gives a representative view of the microbial community. This is very useful when conducting a survey of microbial diversity in an environment, although there is evidence that pooling samples can mask rare species and obscure diversity in patterns in various taxa (Avis et al. 2010, Manter et al. 2010, Engel et al. 2012). In addition, pooled samples may give an inaccurate view of the actual microbial communities and their interactions at fine-scales where the exact composition of the subsamples may be relevant (Alteio et al. 2021, Fleishman et al. 2022).

In the NMDS ordinations (Fig. 2) and clustering analyses (Figure S2, Supporting Information), the composite samples are located centrally, indicating that they represent an average community of European beech deadwood. However, these average communities fail to capture the heterogeneous distribution of deadwood microbes. For example, two fine-scale replicates from sample_049 show a relative abundance of the bacterial genus *Pseudomonas* above 74% (Fig. 3) while, in the third replicate, *Pseudomonas* only makes up 20% of the community. Similarly, the fungal genus *Kretzschmaria* has a relative abundance of at least 85% in two replicates from sample_007 (Fig. 3) but makes up only 63% in the third. This variation is likely to play a major role in accurately identifying microbial associations when using abundance data.

We compared the association networks generated from the composite and fine-scale samples and found evidence that the detected associations change according to sampling scale with networks constructed from fine-scale samples to show much higher levels of natural connectivity. We found that, of the total highly weighted associations observed, 96% of bacterial and 34% of fungal associations would be missed if using composite data. In addition, 1% of bacterial and 17% of fungal associations only occur in the composite data and are likely false associations due to pooling taxa, which are physically separated in the deadwood. This suggests that deadwood studies attempting to show interactions between taxa need to adjust their sampling strategies to reflect heterogeneous deadwood microcommunities.

Fine-scale samples are important for understanding possible microbial interactions as the scales over which most sampling is conducted may be orders of magnitude larger than the scales on which microbes interact (Cordero and Datta 2016, Ladau and Eloe-Fadrosh 2019, Alteio et al. 2021). While the composite deadwood samples were collected from three 40-cm drillings and the fine-scale samples were from a 1-cm^3^ cylinder, recent work has shown that microbial communities at the soil–water interface may stratify at millimetre distances with Gammaproteobacteria decreasing from ∼20% to less than 5% relative abundance over only a 6-mm distance (Cai et al. 2022). Xiong et al. (2022) showed that, even in an overall aerobic environment, fungal hyphae can reduce the oxygen concentration in the surrounding water film at a distance of up to 13 µm from the hyphal surface. These oxygen-depleted microniches provided the conditions necessary for obligate anaerobic bacteria to germinate and grow. Anoxic microniches might play important role in maintaining active diazotrophs in deadwood (Tláskal et al. 2021a). Sampling at the relevant scale for a particular community is necessary to identify the interactions which are possible and the potential functions which are realized.

### Strengths and limitations

As with all studies, there are several limitations and strengths associated with this work. The major strength of this study is the deadwood sampling at a centimetre scale, which allows a much finer grain analysis of microbial communities. However, this also comes with a drawback that material was too limited to perform both DNA extraction and analyse the chemistry of the samples. While we might assume that the chemistry reflects that of the whole deadwood object, there is likely to be variation of the conditions within the deadwood, which may explain the patterns that we observe, e.g. wood density (Rinta-Kanto et al. 2016). Potentially, different bacterial communities could be used as an indicator of the deadwood microenvironment but more sensitive techniques will be needed to test this.

As the sampling is necessarily destructive, we lack information about deadwood microbial dynamics at the centimetre scale and of forest microbial communities more generally (Baldrian 2017). The community may be stable at the scale of the whole deadwood object but, through microbial growth and migration and changing deadwood composition during degradation, there may be substantial variation over time in the fine-scale communities. Unfortunately, addressing this problem will likely require whole new techniques as current sequencing approaches require destructive sampling, preventing studying the dynamics of specific regions, and, as a solid and opaque substance, there is little opportunity for nondestructive monitoring techniques such as fluorescent microscopy in deadwood.

## Conclusion

Our results suggest that fungi are evenly distributed in the deadwood but that bacteria are heterogeneously distributed. The bacterial community detected will likely differ depending on the sampling scale, with variation occurring at the centimetre, and probably even finer, scale. This is of particular importance for studies, which wish to identify microbial associations. Fungi do not show a change in diversity with sampling scale and likely do not form domains, which contain exclusively a single species.

Laboratory-based studies of microbial interactions are extremely valuable but do not reflect the diversity found in the environment and there is a need to confirm laboratory concepts in real-world communities. We also identified large numbers of microbial associations that would be missed or incorrectly identified when using composite data. Care should be taken in association studies where fine-scale, heterogeneous community composition matters more than the average community composition.

We recommend that sampling scale is explicitly considered when designing future experiments, both for deadwood specifically and for environmental microbiology more generally. We agree with previous calls to explicitly consider scale during sampling (Ladau and Eloe-Fadrosh 2019, Alteio et al. 2021, Fleishman et al. 2022) and would recommend pooling samples *in silico* whenever possible as pooling physically samples is irreversible and results in both a loss of fine-scale data and community variability. This may not always be practical due to the amount of time, effort, and/or money required and, in such cases, it is important to properly record the metadata for researchers to understand how the data was sampled in order to assess the validity of different analytical methods.

## Supplementary Material

fiad023_Supplemental_Files

## Data Availability

Raw sequencing data for the composite samples was released as a community resource (Tláskal et al. 2021b) and are available from NCBI BioProject PRJNA672674 (https://www.ncbi.nlm.nih.gov/bioproject/PRJNA672674). The fine-scale sequencing samples are available from NCBI BioProject PRJNA910110 (https://www.ncbi.nlm.nih.gov/bioproject/PRJNA910110/). The sample metadata and scripts used for the analysis are available on Github (https://github.com/jasonbosch/Bacterial-but-not-fungal-communities-show-spatial-heterogeneity-in-European-beech-Fagus-sylvatica).

## References

[bib1] Ahlmann-Eltze C . ggsignif: significance brackets for “ggplot2”. 2019. https://CRAN.R-project.org/package=ggsignif. (last accessed on September 01, 2022).

[bib2] Alteio LV , Séneca J, Canarini A et al. A critical perspective on interpreting amplicon sequencing data in soil ecological research. Soil Biol Biochem. 2021;160:108357.

[bib3] Auguie B . gridExtra: miscellaneous functions for “grid” graphics. 2017. https://CRAN.R-project.org/package=gridExtra. (last accessed on September 01, 2022).

[bib4] Avis PG , Branco S, Tang Y et al. Pooled samples bias fungal community descriptions. Mol Ecol Resour. 2010;10:135–41. 21564998 10.1111/j.1755-0998.2009.02743.x

[bib7] Baldrian P , Zrůstová P, Tláskal V et al. Fungi associated with decomposing deadwood in a natural beech-dominated forest. Fung Ecol. 2016;23:109–22.

[bib5] Baldrian P . Forest microbiome: diversity, complexity and dynamics. Banin E (ed.), FEMS Microbiol Rev. 2016;41:fuw040. doi: 10.1093/femsre/fuw040. 10.1093/femsre/fuw040 27856492

[bib6] Baldrian P . Microbial activity and the dynamics of ecosystem processes in forest soils. Curr Opin Microbiol. 2017;37:128–34. 28689057 10.1016/j.mib.2017.06.008

[bib8] Boddy L . Interspecific combative interactions between wood-decaying basidiomycetes. FEMS Microbiol Ecol. 2000;31:185–94. 10719199 10.1111/j.1574-6941.2000.tb00683.x

[bib9] de Boer W , Folman LB, Summerbell RC et al. Living in a fungal world: impact of fungi on soil bacterial niche development. FEMS Microbiol Rev. 2005;29:795–811. 16102603 10.1016/j.femsre.2004.11.005

[bib10] Bolyen E , Rideout JR, Dillon MR et al. Reproducible, interactive, scalable and extensible microbiome data science using QIIME 2. Nat Biotechnol. 2019;37:852–7. 31341288 10.1038/s41587-019-0209-9 PMC7015180

[bib11] Brabcová V , Nováková M, Davidová A et al. Dead fungal mycelium in forest soil represents a decomposition hotspot and a habitat for a specific microbial community. New Phytol. 2016;210:1369–81. 26832073 10.1111/nph.13849

[bib12] Cai Y-J , Liu Z-A, Zhang S et al. Microbial community structure is stratified at the millimeter-scale across the soil–water interface. ISME Commun. 2022;2:53. 10.1038/s43705-022-00138-z PMC9723559 37938662

[bib13] Callahan BJ , McMurdie PJ, Rosen MJ et al. DADA2: high-resolution sample inference from Illumina amplicon data. Olsson IAS (ed.),Nat Methods. 2016;13:581–3. 27214047 10.1038/nmeth.3869 PMC4927377

[bib14] Caporaso JG , Lauber CL, Walters WA et al. Ultra-high-throughput microbial community analysis on the Illumina HiSeq and MiSeq platforms. ISME J. 2012;6:1621–4. 22402401 10.1038/ismej.2012.8 PMC3400413

[bib15] Carmona-Hernandez S , Reyes-Pérez J, Chiquito-Contreras R et al. Biocontrol of postharvest fruit fungal diseases by bacterial antagonists: a review. Agronomy. 2019;9:121.

[bib16] Charrad M , Ghazzali N, Boiteau V et al. NbClust: an R package for determining the relevant number of clusters in a data set. J Stat Soft. 2014;61:1–36.

[bib17] Christofides SR , Bettridge A, Farewell D et al. The influence of migratory *Paraburkholderia* on growth and competition of wood-decay fungi. Fung Ecol. 2020;45:100937.

[bib18] Cordero OX , Datta MS. Microbial interactions and community assembly at microscales. Curr Opin Microbiol. 2016;31:227–34. 27232202 10.1016/j.mib.2016.03.015 PMC5157693

[bib19] Crowther TW , Boddy L, Jones TH. Outcomes of fungal interactions are determined by soil invertebrate grazers: grazers alter fungal community. Ecol Lett. 2011;14:1134–42. 21929699 10.1111/j.1461-0248.2011.01682.x

[bib20] Deveau A , Bonito G, Uehling J et al. Bacterial–fungal interactions: ecology, mechanisms and challenges. FEMS Microbiol Rev. 2018;42:335–52. 29471481 10.1093/femsre/fuy008

[bib21] Dunnington D . ggspatial: spatial data framework for ggplot2. 2021. https://CRAN.R-project.org/package=ggspatial. (last accessed on September 01, 2022).

[bib22] Engel M , Behnke A, Bauerfeld S et al. Sample pooling obscures diversity patterns in intertidal ciliate community composition and structure. FEMS Microbiol Ecol. 2012;79:741–50. 22093023 10.1111/j.1574-6941.2011.01255.x

[bib23] Eskalen A , Stouthamer R, Lynch SC et al. Host range of Fusarium dieback and its ambrosia beetle (Coleoptera: scolytinae) vector in Southern California. Plant Dis. 2013;97:938–51. 30722538 10.1094/PDIS-11-12-1026-RE

[bib24] Faust K , Sathirapongsasuti JF, Izard J et al. Microbial co-occurrence relationships in the human microbiome. Ouzounis CA (ed.), PLoS Comput Biol. 2012;8:e1002606. 22807668 10.1371/journal.pcbi.1002606 PMC3395616

[bib25] Fleishman SM , Eissenstat DM, Bell TH et al. Functionally-explicit sampling can answer key questions about the specificity of plant–microbe interactions. Environ Microbiome. 2022;17:51. 36221138 10.1186/s40793-022-00445-x PMC9555203

[bib26] FOREST EUROPE . State of Europe's forests 2020. 2020. https://foresteurope.org/state-of-europes-forests/. (last accessed August 10, 2022).

[bib27] Hagge J , Bässler C, Gruppe A et al. Bark coverage shifts assembly processes of microbial decomposer communities in dead wood. Proc R Soc B. 2019;286:20191744. 10.1098/rspb.2019.1744 PMC6790774 31594501

[bib28] Heilmann-Clausen J , Boddy L. Inhibition and stimulation effects in communities of wood decay fungi: exudates from colonized wood influence growth by other species. Microb Ecol. 2005;49:399–406. 16003479 10.1007/s00248-004-0240-2

[bib29] Hiscox J , Boddy L. Armed and dangerous – chemical warfare in wood decay communities. Fung Biol Rev. 2017;31:169–84.

[bib30] Hoppe B , Kahl T, Karasch P et al. Network analysis reveals ecological links between N-fixing bacteria and wood-decaying fungi. Desvaux M (ed.), PLoS ONE. 2014;9:e88141. 24505405 10.1371/journal.pone.0088141 PMC3914916

[bib31] Huber N . ggdendroplot: create dendrograms for ggplot2. 2021. https://github.com/NicolasH2/ggdendroplot. (last accessed September 1, 2022).

[bib32] Ihrmark K , Bödeker ITM, Cruz-Martinez K et al. New primers to amplify the fungal ITS2 region - evaluation by 454-sequencing of artificial and natural communities. FEMS Microbiol Ecol. 2012;82:666–77. 22738186 10.1111/j.1574-6941.2012.01437.x

[bib33] Kleine Bardenhorst S , Vital M, Karch A et al. Richness estimation in microbiome data obtained from denoising pipelines. Comput Struct Biotechnol J. 2022;20:508–20. 35070172 10.1016/j.csbj.2021.12.036 PMC8762370

[bib34] Kõljalg U , Nilsson HR, Schigel D et al. The taxon hypothesis paradigm—on the unambiguous detection and communication of taxa. Microorganisms. 2020;8:1910. 33266327 10.3390/microorganisms8121910 PMC7760934

[bib35] Ladau J , Eloe-Fadrosh EA. Spatial, temporal, and phylogenetic scales of microbial ecology. Trends Microbiol. 2019;27:662–9. 31000488 10.1016/j.tim.2019.03.003

[bib38] McKnight DT , Huerlimann R, Bower DS et al. Methods for normalizing microbiome data: an ecological perspective. Jarman S (ed.), Methods Ecol Evol. 2019;10:389–400.

[bib39] McMurdie PJ , Holmes S. phyloseq: an R package for reproducible interactive analysis and graphics of microbiome census data. Watson M (ed.), PLoS ONE. 2013;8:e61217–. 23630581 10.1371/journal.pone.0061217 PMC3632530

[bib40] McMurdie PJ , Holmes S. Waste not, want not: why rarefying microbiome data is inadmissible. McHardy AC (ed.), PLoS Comput Biol. 2014;10:e1003531. 24699258 10.1371/journal.pcbi.1003531 PMC3974642

[bib36] Maechler M , Rousseeuw P, Struyf A et al. Cluster: cluster analysis basics and extensions. 2021. https://CRAN.R-project.org/package=cluster. ( last accessed September 1, 2022).

[bib37] Manter DK , Weir TL, Vivanco JM. Negative effects of sample pooling on PCR-based estimates of soil microbial richness and community structure. Appl Environ Microbiol. 2010;76:2086–90. 20139317 10.1128/AEM.03017-09 PMC2849261

[bib41] Moll J , Kellner H, Leonhardt S et al. Bacteria inhabiting deadwood of 13 tree species are heterogeneously distributed between sapwood and heartwood: bacteria in deadwood of 13 different tree species. Environ Microbiol. 2018;20:3744–56. 30109768 10.1111/1462-2920.14376

[bib42] Neuwirth E . RColorBrewer: colorBrewer palettes. 2014. https://CRAN.R-project.org/package=RColorBrewer. (last accessed September 1, 2022).

[bib43] Nilsson RH , Larsson K-H, Taylor AFS et al. The UNITE database for molecular identification of fungi: handling dark taxa and parallel taxonomic classifications. Nucleic Acids Res. 2019;47:D259–64. 30371820 10.1093/nar/gky1022 PMC6324048

[bib44] O'Brien SL , Gibbons SM, Owens SM et al. Spatial scale drives patterns in soil bacterial diversity: spatial scale drives soil diversity. Environ Microbiol. 2016;18:2039–51. 26914164 10.1111/1462-2920.13231 PMC4919158

[bib46] O'Leary J , Eastwood D, Müller C et al. Emergent properties arising from spatial heterogeneity influence fungal community dynamics. Fung Ecol. 2018;33:32–9.

[bib45] Oksanen J , Blanchet FG, Friendly M et al. vegan: community ecology package. 2019. https://CRAN.R-project.org/package=vegan. (last accessed September 1, 2022).

[bib47] Paap T , de Beer ZW, Migliorini D et al. The polyphagous shot hole borer (PSHB) and its fungal symbiont fusarium euwallaceae: a new invasion in South Africa. Australasian Plant Pathol. 2018;47:231–7.

[bib48] Pebesma E . Simple features for R: standardized support for spatial vector data. R J. 2018;10:439.

[bib49] Peschel S , Müller CL, von Mutius E et al. NetCoMi: network construction and comparison for microbiome data in R. Briefings Bioinf. 2020;22:bbaa290. 10.1093/bib/bbaa290 PMC8293835 33264391

[bib50] Quast C , Pruesse E, Yilmaz P et al. The SILVA ribosomal RNA gene database project: improved data processing and web-based tools. Nucleic Acids Res. 2013;41:D590–6. 23193283 10.1093/nar/gks1219 PMC3531112

[bib51] R Core Team . R: A Language and Environment for Statistical Computing. Vienna: R Foundation for Statistical Computing, 2022. https://www.R-project.org/

[bib53] Rinta-Kanto JM , Sinkko H, Rajala T et al. Natural decay process affects the abundance and community structure of bacteria and archaea in *Picea abies* logs. de Boer W (ed.), FEMS Microbiol Ecol. 2016;92:fiw087. 27127195 10.1093/femsec/fiw087

[bib52] Rolling T , Zhai B, Frame J et al. Customization of a DADA2-based pipeline for fungal internal transcribed spacer 1 (ITS1) amplicon data sets. JCI Insight. 2022;7. doi: 10.1172/jci.insight.151663. 10.1172/jci.insight.151663 PMC8765055 34813499

[bib54] Seibold S , Müller J, Baldrian P et al. Fungi associated with beetles dispersing from dead wood – let’s take the beetle bus!. Fung Ecol. 2019;39:100–8.

[bib55] Shipunov A , Murrell P, D'Orazio M et al. Shipunov: miscellaneous functions from alexey Shipunov. 2022. https://CRAN.R-project.org/package=shipunov

[bib56] Simonsohn U , Gruson H. Groundhog: the simplest solution to version-control for CRAN packages. 2021. https://CRAN.R-project.org/package=groundhog. ( last accessed September 1, 2022).

[bib57] Slowikowski K . ggrepel: automatically position non-overlapping text labels with “ggplot2”. 2021. https://CRAN.R-project.org/package=ggrepel. (last accessed September 1, 2022).

[bib58] South A . Rnaturalearth: world map data from natural Earth. 2017a. https://CRAN.R-project.org/package=rnaturalearth. (last accessed September 1, 2022).

[bib59] South A . Rnaturalearthdata: world vector map data from natural Earth used in “Rnaturalearth”. 2017b. https://CRAN.R-project.org/package=rnaturalearthdata. ( last accessed September 1, 2022).

[bib60] Steenwyk JL , Rokas A. ggpubfigs: colorblind-friendly color palettes and ggplot2 graphic system extensions for publication-quality scientific figures. Newton ILG (ed.), Microbiol Resour Announc. 2021;10:e00871–21. 10.1128/MRA.00871-21 PMC8567791 34734767

[bib61] Štursová M , Bárta J, Šantrůčková H et al. Small-scale spatial heterogeneity of ecosystem properties, microbial community composition and microbial activities in a temperate mountain forest soil. de Boer W (ed.), FEMS Microbiol Ecol. 2016;92:fiw185. 27604254 10.1093/femsec/fiw185

[bib62] Tláskal V , Brabcová V, Větrovský T et al. Complementary roles of wood-inhabiting fungi and bacteria facilitate deadwood decomposition. Faust K (ed.),Msystems. 2021a;6:e01078–20. 33436515 10.1128/mSystems.01078-20 PMC7901482

[bib63] Tláskal V , Brabcová V, Větrovský T et al. Metagenomes, metatranscriptomes and microbiomes of naturally decomposing deadwood. Sci Data. 2021b;8:198. 34344895 10.1038/s41597-021-00987-8 PMC8333335

[bib64] de Vries A , Ripley BD. Ggdendro: create dendrograms and tree diagrams using “Ggplot2”. 2020. https://CRAN.R-project.org/package=ggdendro. (last accessed September 1, 2022).

[bib65] West JR , Whitman T. Disturbance by soil mixing decreases microbial richness and supports homogenizing community assembly processes. FEMS Microbiol Ecol. 2022;98:fiac089. 35869965 10.1093/femsec/fiac089 PMC9397575

[bib66] Wickham H . ggplot2: Elegant Graphics for Data Analysis. New York: Springer, 2016. https://ggplot2.tidyverse.org. ( last accessed September 1, 2022).

[bib67] Wickham H . stringr: simple, consistent wrappers for common string operations. 2019. https://CRAN.R-project.org/package=stringr. (last accessed September 1, 2022).

[bib68] Willis AD . Rarefaction, alpha diversity, and statistics. Front Microbiol. 2019;10. doi: 10.3389/fmicb.2019.02407. 10.3389/fmicb.2019.02407 PMC6819366 31708888

[bib69] Xiong B-J , Kleinsteuber S, Sträuber H et al. Impact of fungal hyphae on growth and dispersal of obligate anaerobic bacteria in aerated habitats. Bailey MJ (ed.), Mbio. 2022;13:e00769–22. 35638736 10.1128/mbio.00769-22 PMC9239063

[bib70] Yan L . Ggvenn: draw Venn diagram by “Ggplot2”. 2021. https://CRAN.R-project.org/package=ggvenn. (last accessed September 1, 2022).

[bib71] Yilmaz P , Parfrey LW, Yarza P et al. The SILVA and “All-species Living Tree Project (LTP)” taxonomic frameworks. Nucl Acids Res. 2014;42:D643–8. 24293649 10.1093/nar/gkt1209 PMC3965112

[bib72] Zhang L , Xu M, Liu Y et al. Carbon and phosphorus exchange may enable cooperation between an arbuscular mycorrhizal fungus and a phosphate-solubilizing bacterium. New Phytol. 2016;210: 1022–32. 27074400 10.1111/nph.13838

