# Supplementary material for: Bacterial, but not fungal, communities show spatial heterogeneity in European beech (Fagus sylvatica L.) deadwood
Source: FEMS Microbiol Ecol. 2023 Mar 11;99(4):fiad023. doi: 10.1093/femsec/fiad023 (PMC10065134; doi:10.1093/femsec/fiad023)
Supplement: fiad023_Supplemental_Files [file fiad023_supplemental_files.zip › Supp_data_Figure_Legends.docx]

### Supplementary Figure Legends

Figure S1: Map showing the location of Žofínský Prales National Nature Reserve (red) in the Czech Republic. Major roads (grey) and cities (black) are also indicated.

Figure S2: Clustering of bacterial (A) and fungal (C) communities according to the quantitative Jaccard distances between them. The support for each branch point is given as the percentage of times a branch point appeared in 1000 bootstrap replications. Except for the terminal branches, the length of the branches is proportionate the distance between the samples. The sample names are coloured according to the deadwood object, as in other figures, while the branches are coloured to show the optimal clusters. Fine-scale samples are labelled with A-C while composite samples are labelled with X. The estimated optimal number of clusters was determined by the most commonly-returned number of clusters from a set of 20 clustering indices and is shown as a histogram for bacterial (B) and fungal (D) communities.

Figure S3: Non-metric Multidimensional Scaling (NMDS) plots of the bacterial and fungal communities based on Hellinger distances at the ASV level.

Figure S4: Stacked bar charts showing the taxonomic composition of the different samples at the phylum (A, B) and order (C, D) levels for bacteria (A, C) and fungi (B, D). Only ASVs with a mean relative abundance greater than 1% are identified, all ASVs with a mean relative abundance less than 1% are included in the grey ‘Rare_Taxa’ category.

Figure S5: Comparison of composite and fine-scale association networks. Networks were constructed with CCREPE. Positive associations are shown by blue edges and negative associations are shown by red edges. Nodes coloured per network according the cluster in which they fall. Highly transparent edges are those below the maximum of the 75th quantile of absolute edge strengths or the edge strength corresponding to 2n-th edge strength.
