# Supplementary figures and images for: Bacterial, but not fungal, communities show spatial heterogeneity in European beech (Fagus sylvatica L.) deadwood
Source: FEMS Microbiol Ecol. 2023 Mar 11;99(4):fiad023. doi: 10.1093/femsec/fiad023 (PMC10065134; doi:10.1093/femsec/fiad023)

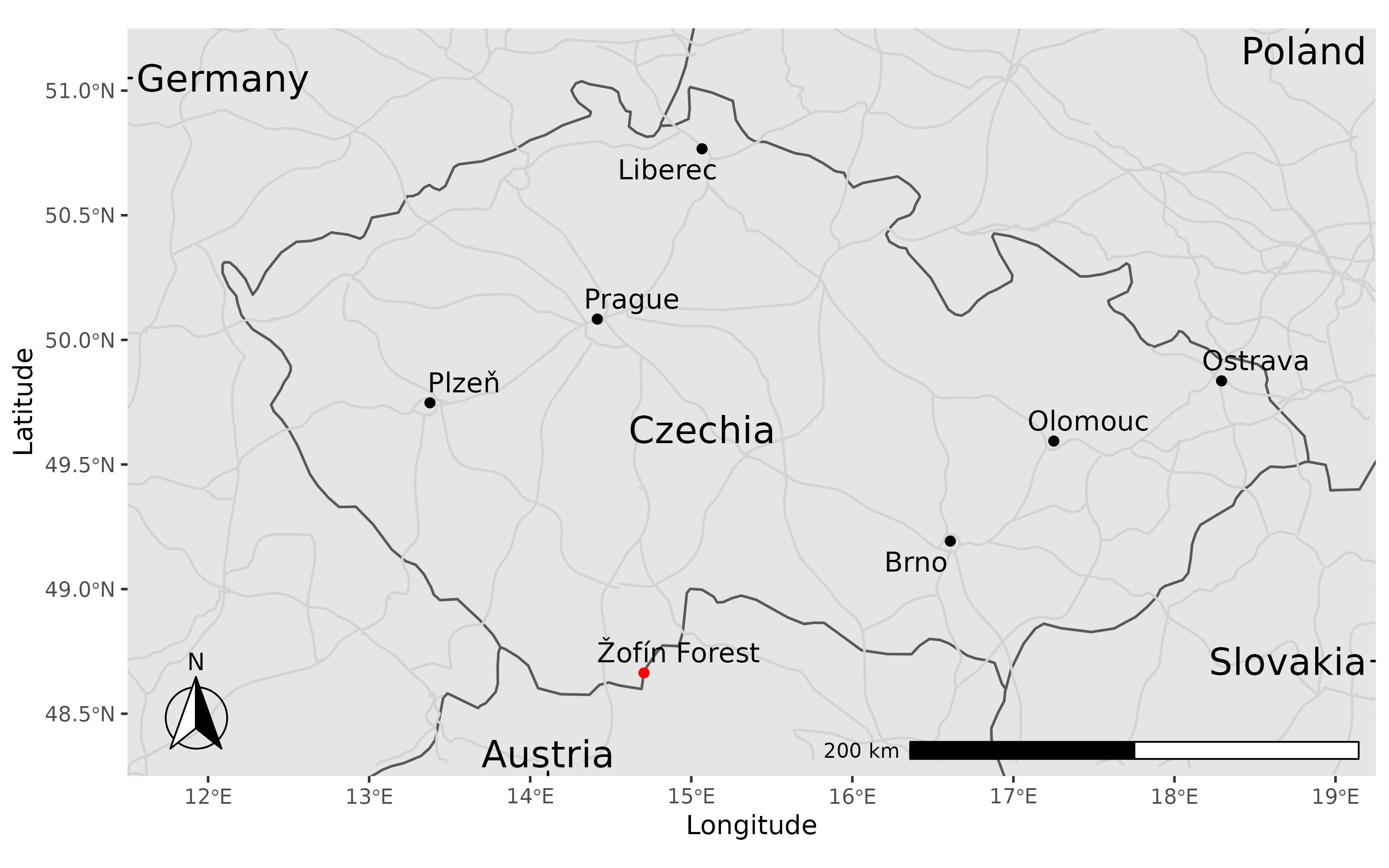

Supplement: fiad023_Supplemental_Files [file fiad023_supplemental_files.zip › Figure_S1_Supplementary_Data.jpg]

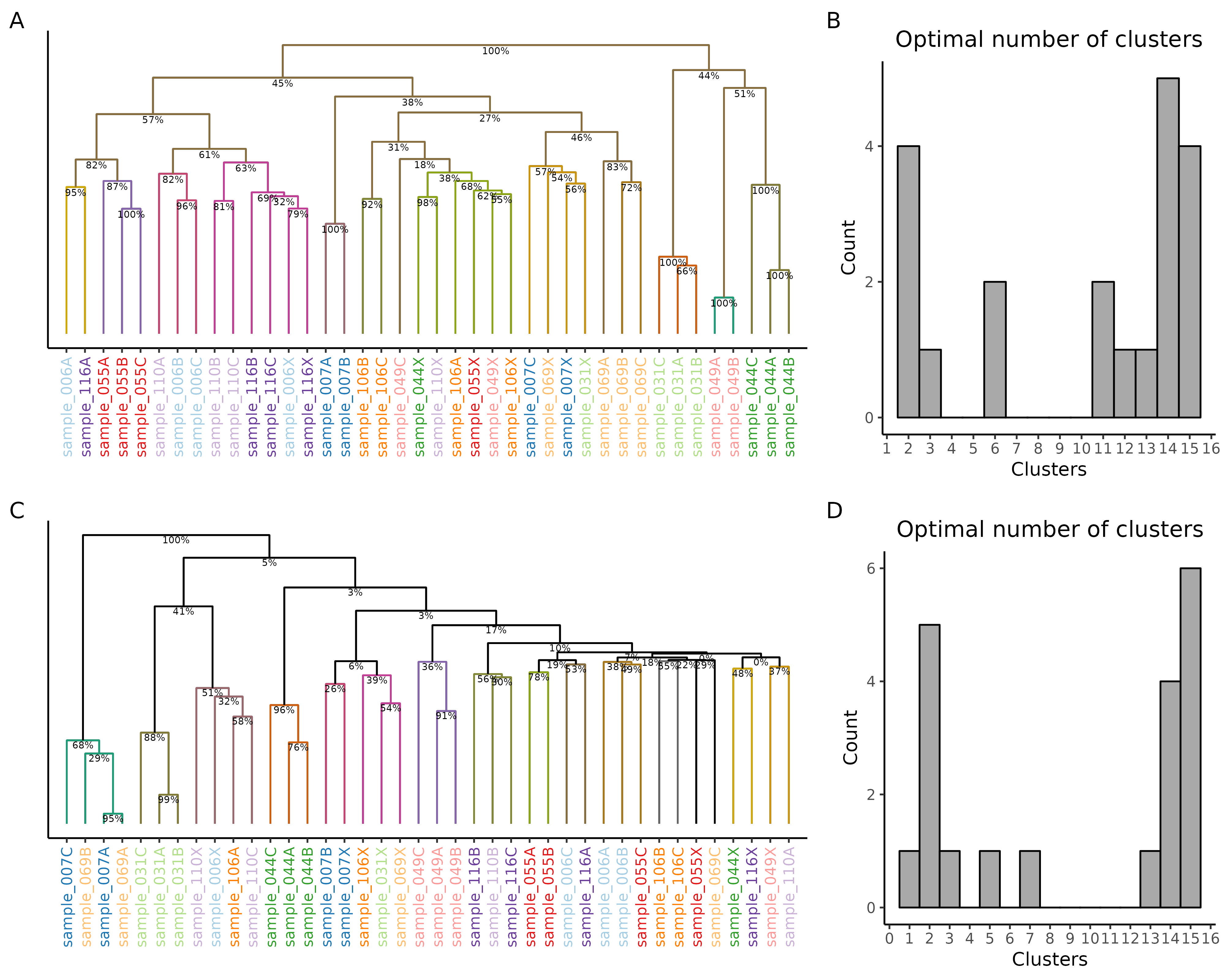

Supplement: fiad023_Supplemental_Files [file fiad023_supplemental_files.zip › Figure_S2_Supplementary_Data.jpg]

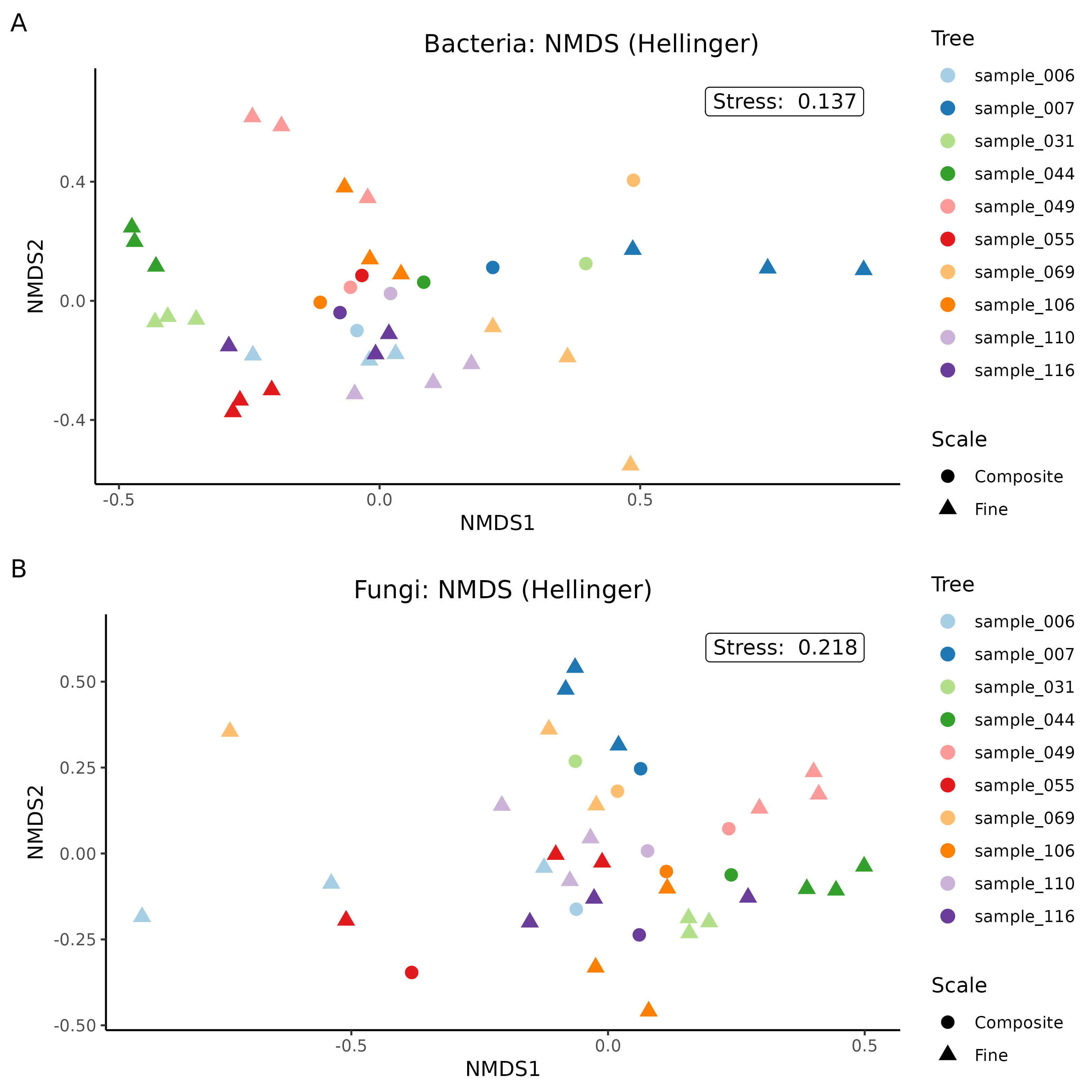

Supplement: fiad023_Supplemental_Files [file fiad023_supplemental_files.zip › Figure_S3_Supplementary_Data.jpg]

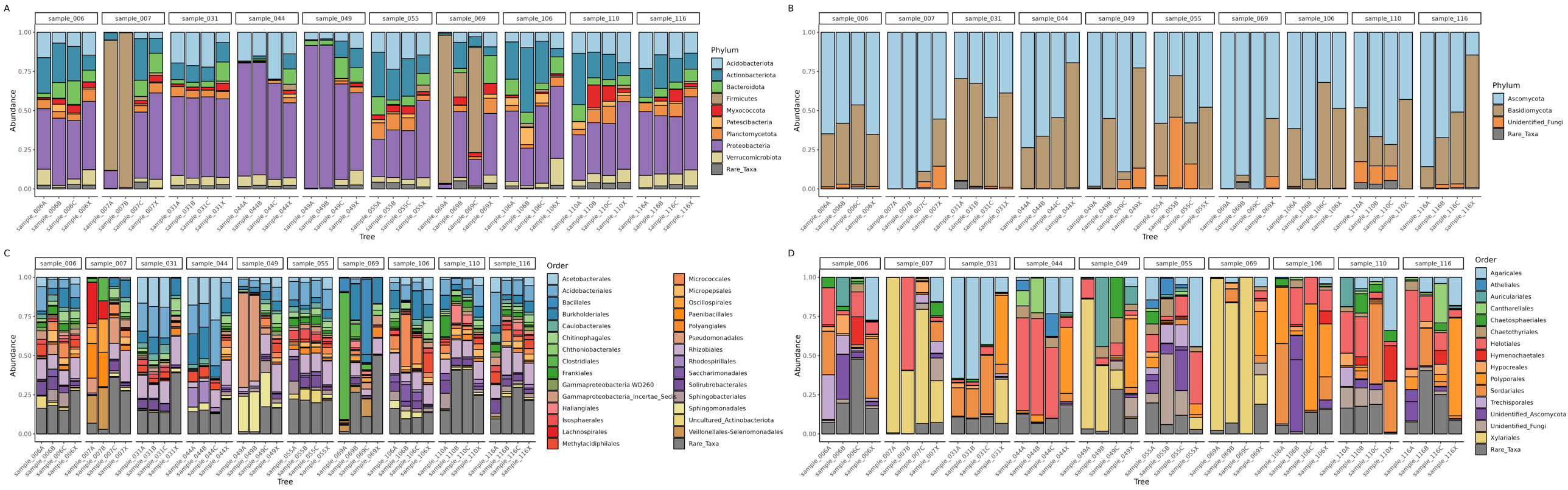

Supplement: fiad023_Supplemental_Files [file fiad023_supplemental_files.zip › Figure_S4_Supplementary_Data.jpg]

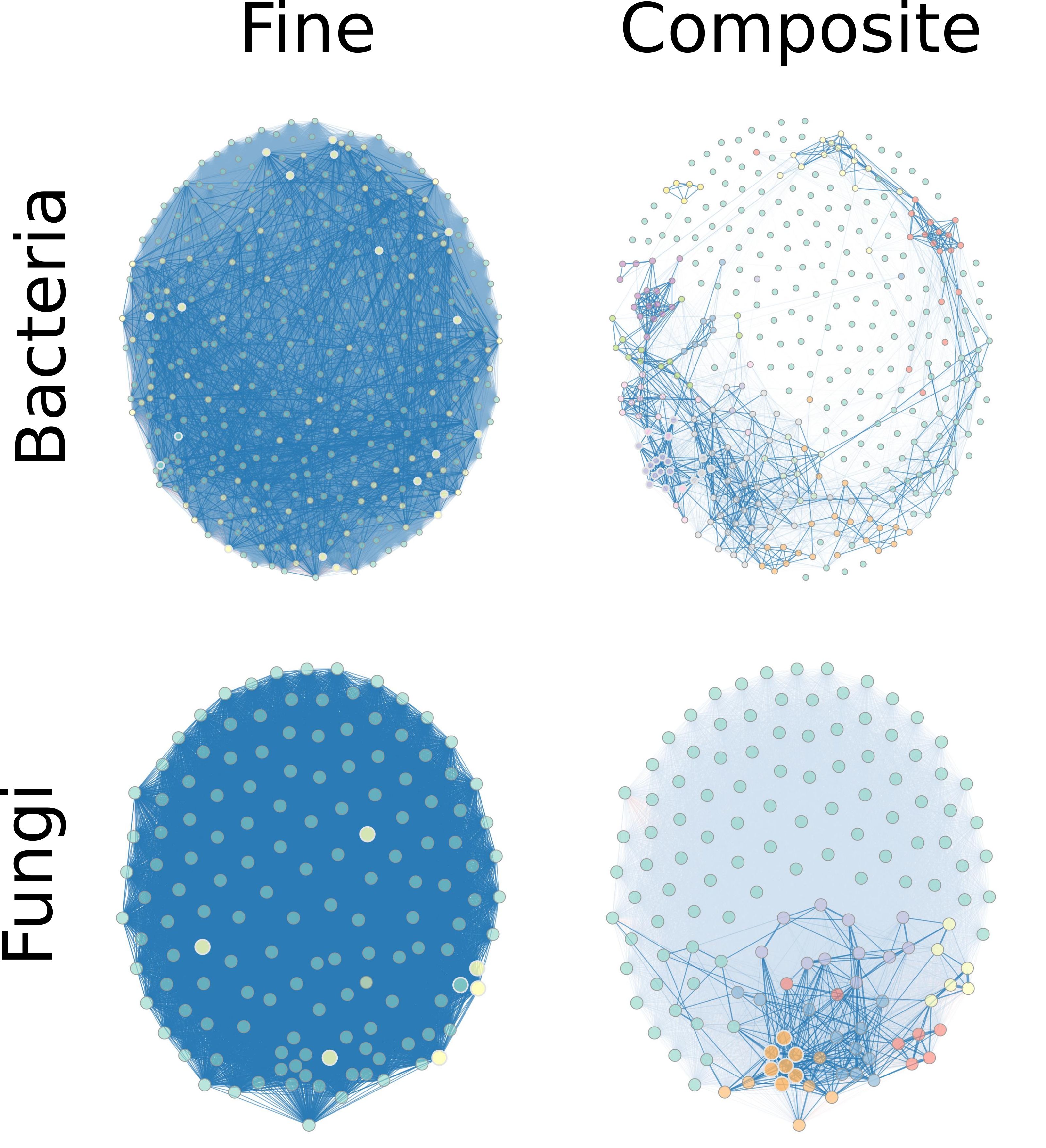

Supplement: fiad023_Supplemental_Files [file fiad023_supplemental_files.zip › Figure_S5_Supplementary_Data.jpg]
